# Addressing Profiles of Systemic Inflammation Across the Different Clinical Phenotypes of Acutely Decompensated Cirrhosis

**DOI:** 10.3389/fimmu.2019.00476

**Published:** 2019-03-19

**Authors:** Jonel Trebicka, Alex Amoros, Carla Pitarch, Esther Titos, José Alcaraz-Quiles, Robert Schierwagen, Carmen Deulofeu, Javier Fernandez-Gomez, Salvatore Piano, Paolo Caraceni, Karl Oettl, Elsa Sola, Wim Laleman, Jane McNaughtan, Rajeshwar P. Mookerjee, Minneke J. Coenraad, Tania Welzel, Christian Steib, Rita Garcia, Thierry Gustot, Miguel A. Rodriguez Gandia, Rafael Bañares, Agustin Albillos, Stefan Zeuzem, Victor Vargas, Faouzi Saliba, Frederic Nevens, Carlo Alessandria, Andrea de Gottardi, Heinz Zoller, Pere Ginès, Tilman Sauerbruch, Alexander Gerbes, Rudolf E. Stauber, Mauro Bernardi, Paolo Angeli, Marco Pavesi, Richard Moreau, Joan Clària, Rajiv Jalan, Vicente Arroyo

**Affiliations:** ^1^European Foundation for the Study of Chronic Liver Failure, Barcelona, Spain; ^2^Department of Internal Medicine I, University of Bonn, Bonn, Germany; ^3^Faculty of Health Sciences, University of Southern Denmark, Odense, Denmark; ^4^Department of Mechanical Biology, Institute for Bioengineering of Catalonia, Barcelona, Spain; ^5^J.W. Goethe University Hospital, Frankfurt, Germany; ^6^Department of Biochemistry and Molecular Genetics, Hospital Clínic, IDIBAPS and CIBERehd, Barcelona, Spain; ^7^Liver Unit, Hospital Clínic, IDIBAPS and CIBERehd, Barcelona, Spain; ^8^Unit of Internal Medicine and Hepatology, Department of Medicine, DIMED, University of Padova, Padova, Italy; ^9^Department of Medical and Surgical Sciences, University of Bologna, Bologna, Italy; ^10^Department of Gastroenterology and Hepatology, Medical University of Graz, Graz, Austria; ^11^University Hospital Gasthuisberg, KU Leuven, Leuven, Belgium; ^12^Royal Free Hospital, London, United Kingdom; ^13^Department of Gastroenterology and Hepatology, Leiden University Medical Center, Leiden, Netherlands; ^14^Department of Medicine II, Liver Center Munich, University Hospital LMU Munich, Munich, Germany; ^15^Department of Digestive Diseases and CIBERehd, Facultad de Medicina, Hospital General Universitario Gregorio Marañón, Instituto de Investigación Sanitaria Gregorio Marañón, Universidad Complutense, Madrid, Spain; ^16^Erasme Hospital, Université Libre de Bruxelles, Brussels, Belgium; ^17^Hospital Ramón y Cajal, Madrid, Spain; ^18^Vall'd Hebron Hospital, Barcelona, Spain; ^19^Hôpital Paul Brousse, Université Paris-Sud, Villejuif, France; ^20^Division of Gastroenterology and Hepatology, San Giovanni Battista Hospital, Torino, Italy; ^21^Department of Hepatology, Inselspital, Bern, Switzerland; ^22^Department of Hepatology and Gastroenterology, University Clinic Innsbruck, Innsbruck, Austria; ^23^Inserm, U1149, Centre de Recherche sur l'Inflammation (CRI), UMRS1149, Paris, France; ^24^Université Paris Diderot-Paris 7, Département Hospitalo-Universitaire (DHU) UNITY, Paris, France; ^25^Service d'Hépatologie, Hôpital Beaujon, Assistance Publique-Hôpitaux de Paris, Paris, France; ^26^Laboratoire d'Excellence Inflamex, ComUE Sorbonne Paris Cité, Paris, France

**Keywords:** acute decompensation, cirrhosis, signature, ACLF, organ failure, organ dysfunction

## Abstract

**Background:** Patients with acutely decompensated cirrhosis (AD) may or may not develop acute-on-chronic liver failure (ACLF). ACLF is characterized by high-grade systemic inflammation, organ failures (OF) and high short-term mortality. Although patients with AD cirrhosis exhibit distinct clinical phenotypes at baseline, they have low short-term mortality, unless ACLF develops during follow-up. Because little is known about the association of profile of systemic inflammation with clinical phenotypes of patients with AD cirrhosis, we aimed to investigate a battery of markers of systemic inflammation in these patients.

**Methods:** Upon hospital admission baseline plasma levels of 15 markers (cytokines, chemokines, and oxidized albumin) were measured in 40 healthy controls, 39 compensated cirrhosis, 342 AD cirrhosis, and 161 ACLF. According to EASL-CLIF criteria, AD cirrhosis was divided into three distinct clinical phenotypes (AD-1: Creatinine<1.5, no HE, no OF; AD-2: creatinine 1.5–2, and or HE grade I/II, no OF; AD-3: Creatinine<1.5, no HE, non-renal OF).

**Results:** Most markers were slightly abnormal in compensated cirrhosis, but markedly increased in AD. Patients with ACLF exhibited the largest number of abnormal markers, indicating “full-blown” systemic inflammation (all markers). AD-patients exhibited distinct systemic inflammation profiles across three different clinical phenotypes. In each phenotype, activation of systemic inflammation was only partial (30% of the markers). Mortality related to each clinical AD-phenotype was significantly lower than mortality associated with ACLF (*p* < 0.0001 by gray test). Among AD-patients baseline systemic inflammation (especially IL-8, IL-6, IL-1ra, HNA2 independently associated) was more intense in those who had poor 28-day outcomes (ACLF, death) than those who did not experience these outcomes.

**Conclusions:** Although AD-patients exhibit distinct profiles of systemic inflammation depending on their clinical phenotypes, all these patients have only partial activation of systemic inflammation. However, those with the most extended baseline systemic inflammation had the highest the risk of ACLF development and death.

## Introduction

Natural history of patients with acutely decompensated (AD) cirrhosis may be complicated by acute-on-chronic liver failure (ACLF) (1). ACLF, which has been intensively investigated during the recent years, is characterized by the presence of organ failure(s) (OFs) and high short-term mortality (1–4). The diagnosis of OFs is based on the CLIF-C OF scoring system which assesses the deterioration in the function of the six major organ systems, including liver, kidney, coagulation, brain, circulation, and respiration (1). ACLF is recognized when patients have either a single renal failure; moderate renal dysfunction (creatinine between 1.5 and 1.9 mg/dl) and/or cerebral dysfunction (grade I and II hepatic encephalopathy) in combination with any isolated non-renal OF; or two OFs or more (1). ACLF is also characterized by the presence of high-grade systemic inflammation. Many biomarkers of systemic inflammation are elevated in ACLF, and associated with outcome (5–12).

Unlike patients with ACLF, patients with AD have low short-term mortality (1). AD-patients without ACLF at hospital admission may present three distinct clinical phenotypes which do no overlap (1). The first phenotype (hereafter called AD-1) includes patients without any single OF, who have serum creatinine of <1.5 mg/dL and do not have hepatic encephalopathy (HE). The second phenotype (AD-2) includes patients with isolated renal dysfunction and/or HE I or II, but without any associated single non-renal OF. Finally, the third phenotype (AD-3) includes patients with a single non-renal OF without any kidney dysfunction. Although it is known that some AD-patients without ACLF at hospital admission can subsequently develop ACLF and die (1), the baseline profile of systemic inflammation in these patients developing or not ACLF during short-term follow-up is unknown. Also the profiles of systemic inflammation across the three distinct clinical phenotypes have not been investigated. Expanding our knowledge about the profile of systemic inflammation associated with each clinical phenotype should deliver not only insights into the pathogenesis of ACLF, and also provide clinical tools for stratification of patients and therapy (e.g., anti-TNF, G-CSF).

We hypothesized that each of the three distinct clinical phenotypes which compose the group of patients with AD cirrhosis who were free of ACLF may have a distinctive baseline inflammatory profile. In addition, we wondered whether, among the patients with AD cirrhosis without ACLF, the baseline inflammatory profile was able to distinguish those who will develop ACLF during follow-up from who will not, as well as differentiate those who will die from those who will remain alive. To address these hypotheses, we investigated a battery of markers of systemic inflammation in a large cohort of 582 individuals including healthy controls, patients with compensated cirrhosis without prior decompensation, patients with AD who were free of ACLF, and patients with ACLF.

## Patients and Methods

### Patients

In all patients, presence of cirrhosis was diagnosed either by unequivocal signs in imaging, presence of complications of portal hypertension or development of AD and/or ACLF. This study analyzed a total of 582 individuals, of whom 542 were patients with cirrhosis. Three hundred and forty-two of these had been enrolled in the CANONIC study and were selected because they had AD cirrhosis but no ACLF at enrollment (1). These 342 patients were compared to 39 patients with compensated cirrhosis who had never presented an episode of decompensation, and 40 healthy volunteers as negative controls. Moreover, 161 patients with ACLF (95 ACLF grade 1, 66 patients with ACLF grade 2) enrolled in the CANONIC study were selected to serve as positive controls, since those patients have an extensive elevation of all systemic inflammation markers. The selection of the CANONIC study patients was based on the availability of blood samples within the first 2 days after enrollment from patients under intensive surveillance during hospitalization (5). All patients gave their written informed consent. Each center obtained the ethics approval from the local ethics committee for the CANONIC study (1, 5).

### Definition of AD Cirrhosis, OF, and ACLF

AD of cirrhosis was defined according to criteria established by the CANONIC study (1). Briefly, it includes acute development of large ascites, hepatic encephalopathy, gastrointestinal hemorrhage, bacterial infection, or any combination of these (1).

Individual OFs were diagnosed according to the CLIF-C OF score (1). Liver failure was defined by serum bilirubin of 12 mg/dl or more, kidney failure by creatinine of 2 mg/dl or more (or renal replacement therapy), coagulation failure by INR of 2.5 or more. Circulatory failure was diagnosed when vasopressors were used, and respiratory failure when the patient received mechanical ventilation (not due to HE-induced coma) or PaO_2_/FiO_2_ was 200 or lower. Finally, cerebral failure was defined as HE grade III and IV (1).

As mentioned earlier, three distinct phenotypes characterized of patients with AD without ACLF at admission, and ACLF was defined according to criteria established by the CANONIC study (1).

### Data Collection

Healthy controls were recruited among 45–65 year-old medical and non-medical staff from the Hospital Clinic, while patients with compensated cirrhosis were recruited from the University Hospital Bologna, University Hospital Padova and Royal Free Hospital London and the data at baseline were recorded. Data from the CANONIC study patients were obtained as previously described (1, 5). Briefly, data from previous medical history, physical examination, and laboratory parameters were recorded at baseline, including etiology, previous episodes of acute decompensation, potential precipitating events and reason for hospitalization. Moreover, close 28-day follow-up data were collected according to the CANONIC protocol (1). Finally, information on liver transplantation, mortality and causes of death were obtained on day 28, and at 3 and 6 months and 1 year after enrollment.

### Sample Collection and Analysis of biomarkers

The baseline blood samples were obtained in Vacutainer EDTA tubes at the time of enrollment in the study and/or within the first 2 days after enrolment in the study (48 h of hospital admission). Samples at the last assessment could be obtained in 132 patients. In all cases, blood was rapidly centrifuged at 4°C and the plasma frozen at −80°C until analysis.

We measured TNF-α, IL-6, IL-8, MCP-1, IP-10, MIP-1β, G-CSF, GM-CSF, IL-10, IL-1ra, INFγ, IL-17A, IL-7, and eotaxin in 25 μl of plasma using a multiplexed bead-based immunoassay (Milliplex MAP Human Cytokine/Chemokine Magnetic Bead Panel (Merck Millipore, Darmstadt, Germany) on a Luminex 100 Bioanalyzer (Luminex Corp., Austin, TX). The readouts were analyzed with the standard version of the Milliplex Analyst software (Merck Millipore). A five-parameter logistic regression model was used to create standard curves (pg/mL) and to calculate the concentration of each sample. Finally, the levels of irreversibly oxidized albumin (HNA2) were assessed by high performance liquid chromatography (5) as marker of systemic oxidative stress. The levels of systemic inflammation markers in patients with ACLF have been published previously (5).

### Statistical Analysis

Plasma levels were above detection limits in most patients. In healthy subjects and patients with values of cytokines or any other measurement below the detection limit, the threshold of detection was assigned as the determined value. Results are presented as frequencies and percentages for categorical variables, means and SDs for normally distributed continuous variables and medians with interquartile range for not normally distributed continuous variables. Hierarchical clustering analysis was performed using the GP plot package from R software. Intensity of inflammation was evaluated according to the relationship between the set of cytokines in different combinations stratifying for different groups of patients. In univariate statistical comparisons, Chi-square test was used for categorical variables, Student's *t*-test or ANOVA for normal continuous variables and Mann-Whitney *U*-test or Kruskal-Wallis test for non-normal continuous variables. Multiple-testing was corrected by the Bonferroni correction (corrected *p*-value for 15 markers 0.05/15 = 0.003). To assess the strength of the association between each marker and ACLF, logistic regression models were performed. Factors showing a clinically and statistically significant association to the outcome in univariate analyses were selected for the initial model. The final models were fitted using a stepwise forward method based on likelihood ratios with the same significance level (*p* < 0.05) for entering and dropping variables. The proportional hazards model for competing risks proposed by Fine and Gray was used to identify independent predictors of mortality as previously described (1). This model was chosen to account for liver transplantation as an event “competing” with mortality. Variables with a skewed distribution were log-transformed for statistical analyses and graphical comparisons. A *p*- ≤ 0.05 was considered statistically significant. Analyses were done with SPSS V. 23.0, SAS V.9.4, and R V.3.4.2 statistical packages.

## Results

### General Characteristics of the Patients

This study investigated 15 markers of systemic inflammation and oxidative stress in 342 AD-patients but without ACLF at admission. These were compared to the levels measured in 161 patients admitted to the hospital with ACLF grade 1 or 2, 39 patients with compensated cirrhosis and no prior decompensation episode, and 40 healthy controls (Supplementary Tables 1,2). The reason for selecting only patients with ACLF grade 1 or 2 was to exclude severely diseased patients who had three OFs or more, since the enormous elevation of inflammatory markers in these patients may make difficult the comparison of their profile of systemic inflammation with that of patients with AD and without ACLF.

Importantly, our patients with compensated cirrhosis had never experienced any decompensation, despite the fact that these patients were at risk of developing it. Briefly, these patients had a mean value of 37.8 kPa (21.4–49.7 kPa) measured by Fibroscan^®^ (Echosense, France) and median platelet count of 108 x 10^9^/L (72–159 × 10^9^/L), surrogates suggesting the presence of clinical significant portal hypertension (13). Moreover, in 18 (46%) patients, esophageal varices were already diagnosed. Of note, levels of systemic inflammation markers were only moderately altered in patients with compensated cirrhosis compared to healthy controls (Supplementary Table 1), indicating the absence of significant systemic inflammation in most of these patients. Of note, patients with compensated cirrhosis were analyzed only in a cross-sectional manner, precluding any assessment of the development of AD disease in these patients (Supplementary Table 1).

While the demography was similar, there were important, but expected between-group differences, with the most abnormal values being observed in the ACLF group (Supplementary Table 2).

### Markers of Systemic Inflammation According to the Three Clinical Phenotypes in AD Patients

**Figure 1:**
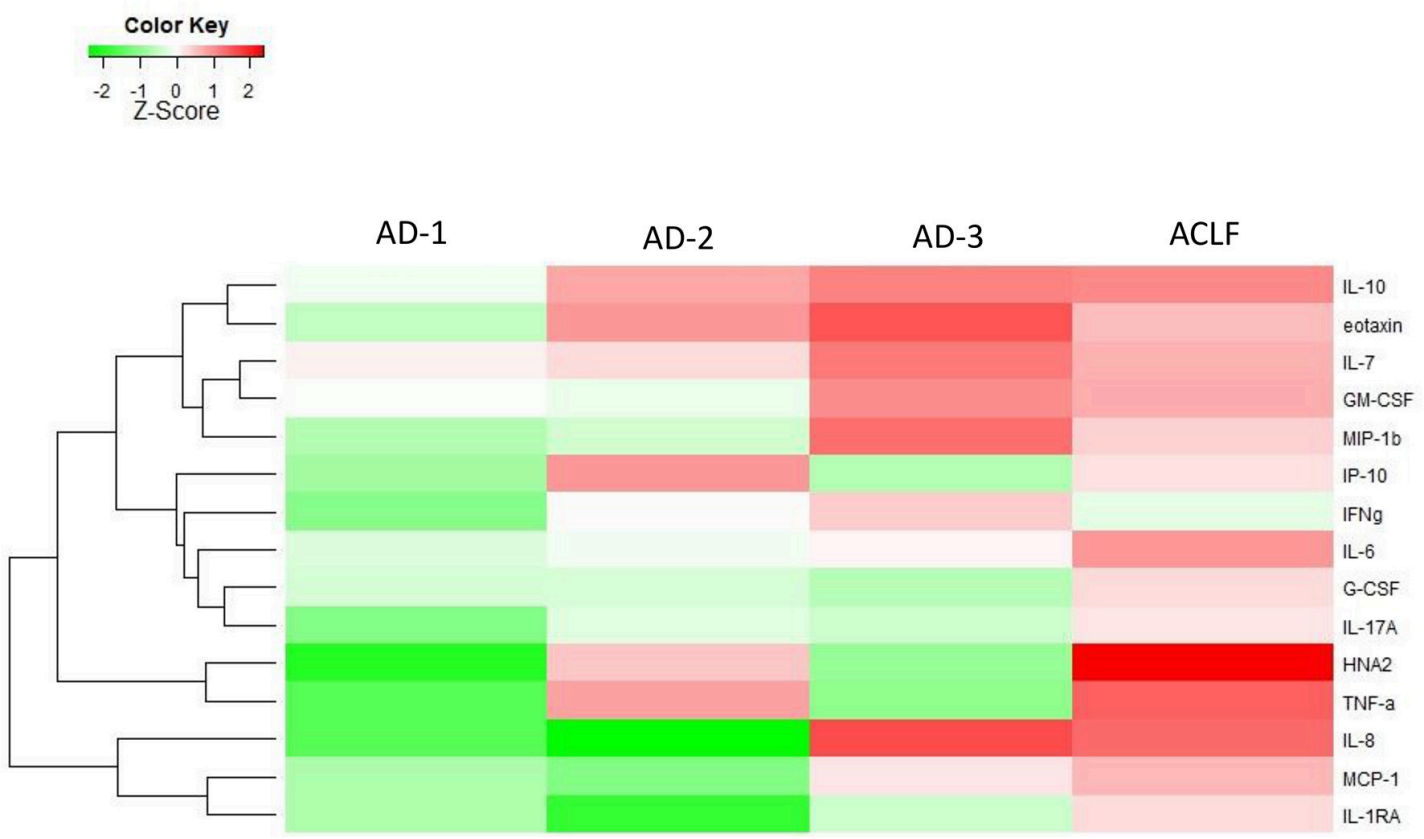
Heat-map highlighting medians of the levels of the different biomarkers of systemic inflammation in patients with acutely decompensated (AD) cirrhosis (with and without ACLF). The patients with “ACLF-free” AD cirrhosis were stratified into three phenotypes. The first phenotype (AD-1) included patients without any single OF, who have serum creatinine of <1.5 mg/dL and do not have hepatic encephalopathy. The second phenotype (AD-2) included patients with isolated renal dysfunction and/or cerebral dysfunction, i.e., without any associated single non-renal, non-cerebral OF. The third phenotype (AD-3) included patients with a single non-renal OF, without any kidney dysfunction. The magnitude of the levels is color-coded and the clustering for each marker with the rest of the markers is shown to the left of the heat-map.

The profile of systemic inflammation markers significantly differed across the three phenotypes of AD without ACLF (AD-1, AD-2, and AD-3; Figure 1, Table 1 depicting median values). Interestingly, lower levels of TNF-α (OR, 0.52; 95%-CI, 0.34–0.79), eotaxin (OR, 0.57; 95% CI, 0.38–0.86) and HNA2 (OR, 0.64; 95% CI, 0.45–0.91) were independently associated with AD-1, while higher levels of TNF-α (OR, 3.25; 95% CI, 2.00–5.28) and HNA2 (OR, 1.75; 95% CI, 1.20–2.55) but lower levels of IL-8 (OR, 0.67; 95% CI, 0.53–0.85) were independently associated with AD-2 (renal and/or cerebral dysfunction, Supplementary Table 3). By contrast, higher levels of IL-8 (OR, 2.30; 95% CI, 1.72–3.06) and lower levels of G-CSF (OR, 0.78; 95% CI, 0.64–0.94) were independently associated with isolated non-renal OF (AD-3, Supplementary Table 3). Importantly, all these results were independent of presence of infection, since we could not find any association of those markers with the presence of infection in the respective stratification of the patients, while IL-6 was independently associated with infection in the entire cohort (OR, 1.36; 95%-CI 1.13–1.65; *p* = 0.01).

**Table 1:** Clinical characteristics, routine laboratory tests, and inflammatory mediators, all at enrollment, across the three distinct phenotypes of patients with acutely decompensated (AD) cirrhosis who were free of ACLF (AD-1, AD-2, AD-3), and the group of patients with ACLF.

**Variable**	**AD-1**	**AD-2**	**AD-3**	**ACLF**	***P*-value**
	**(*N* = 155)**	**(*N* = 121)**	**(*N* = 66)**	**(*N* = 161)**	
**CLINICAL CHARACTERISTICS**					
Age—year	57.6 ± 11.90	59.2 ± 10.35	51.7 ± 11.25	57.3 ± 11.45	< 0.001
Male gender—no./total no. (%)	99/155 (63.9)	83/121 (68.6)	45/66 (68.2)	108/161 (67.1)	0.843
Mean arterial pressure—mm Hg	84.1 ± 10.87	83.1 ± 13.02	84.9 ± 10.63	80.8 ± 13.40	0.050
**PRECIPITATING EVENTS—NO./TOTAL NO. (%)**					
Alcohol consumption	19/144 (13.2)	7/112 (6.3)	14/59 (23.7)	27/150 (18.0)	0.008
Mortality at 90 days	22/155 (14.2)	21/121 (17.4)	12/66 (18.2)	59/161 (36.6)	< 0.001
**ETIOLOGY OF CIRRHOSIS—NO./TOTAL NO. (%)**					
Alcoholic	66/145 (45.5)	55/113 (48.7)	36/63 (57.1)	90/152 (59.)	0.080
HCV	40/145 (27.6)	28/113 (24.8)	10/63 (15.9)	27/152 (17.8)	0.109
Alcohol ± HCV	11/145 (7.6)	13/113 (11.5)	5/63 (7.9)	17/152 (11.2)	0.623
Others	28/145 (19.3)	17/113 (15.0)	12/63 (19.0)	18/152 (11.8)	0.300
**MEDIAN VALUES FOR ROUTINE LABORATORY TESTS (IQR)**					
Serum albumin—g/dl	2.9 (2.50–3.25)	2.9 (2.60–3.30)	2.8 (2.40–3.10)	3.0 (2.40–3.40)	0.157
Serum bilirubin—mg/d	2.5 (1.49–5.18)	2.8 (1.56–5.20)	13.7 (4.90–22.30)	6.1 (2.00–14.37)	< 0.001
Serum creatinine—mg/dl	0.8 (0.66–1.00)	1.5 (0.98–1.70)	0.8 (0.70–1.05)	2.2 (0.98–3.04)	< 0.001
C-reactive protein—mg/L	16.7 (6.80–41.40)	17.9 (6.50–43.00)	19.9 (6.50–34.00)	25.0 (9.70–50.40)	0.163
International Normalized Ratio	1.5 (1.27–1.73)	1.5 (1.27–1.66)	1.7 (1.39–2.20)	1.7 (1.37–2.30)	< 0.001
Platelets— × 10^9^/L	95.0 (63.00–131.00)	84.5 (53.50–139.50)	81.0 (43.00–135.00)	76.0 (53.00–121.00)	0.094
White-cell count— × 1 0^9^/L	6.5 (4.38–9.88)	6.1 (4.60–7.90)	6.6 (4.08–10.72)	8.0 (5.30–12.20)	< 0.001
**MEDIAN VALUES FOR INFLAMMATORY MEDIATORS (IQR)**					
TNF-α—pg/ml	17.9 (13.43–26.64)	24.2 (17.88–34.23)	19.9 (13.05–29.26)	29.0 (17.38–42.83)	< 0.001
IL-6—pg/ml	21.2 (11.71–44.47)	25.0 (12.75–54.20)	24.6 (14.49–47.12)	36.7 (13.79–106.83)	< 0.001
IL-8—pg/ml)	35.9 (19.45–75.22)	37.3 (21.84–63.59)	78.4 (41.69–220.92)	84.5 (38.64–165.10)	< 0.001
MCP-1—pg/ml	316.8 (235.9–395.7)	323.1 (209.49–455.92)	372.2 (254.60–494.73)	410.3 (293.88–690.00)	< 0.001
IP-10—pg/ml	904.6 (530.4–1499.0)	1200.0 (627.0–2255.0)	950.3 (639.9–1718.0)	1147.0 (651.2–2123.0)	0.022
MIP-1ß—pg/ml	20.1 (13.55–37.43)	22.6 (12.93–34.30)	30.2 (16.88–45.41)	26.2 (17.89–42.55)	0.002
G-CSF—pg/ml	22.6 (12.32–54.85)	24.0 (11.19–52.24)	23.0 (12.80–46.65)	30.5 (13.85–81.63)	0.186
GM-CSF—pg/ml	5.0 (2.28–9.08)	4.5 (1.75–10.98)	7.5 (3.68–15.87)	6.8 (3.47–15.97)	0.003
IL-10—pg/ml	3.0 (0.90–9.36)	4.0 (1.37–13.07)	5.3 (1.88–13.39)	7.2 (1.90–25.78)	< 0.001
IL1-ra—pg/ml	11.9 (5.40–25.90)	10.4 (4.52–23.03)	13.4 (6.14–40.97)	18.7 (8.56–50.48)	< 0.001
IFNγ—pg/ml	4.4 (1.65–19.38)	6.7 (2.38–24.29)	7.8 (2.82–18.85)	6.0 (2.32–23.12)	0.107
Eotaxin—pg/ml	103.7 (68.43–152.08)	120.4 (90.48–162.49)	137.0 (94.67–173.76)	123.5 (86.55–177.21)	0.008
IL-17A—pg/ml	3.1 (1.37–13.10)	4.2 (2.03–10.00)	3.8 (1.75–11.31)	4.8 (1.62–14.90)	0.376
IL-7—pg/ml	2.5 (0.85–7.46)	2.7 (1.13–11.74)	4.3 (1.38–8.80)	3.5 (1.62–11.07)	0.135
HNA2—%	4.2 (2.35–8.07)	6.9 (3.79–10.99)	5.6 (3.07–8.85)	11.0 (6.25–15.15)	< 0.001

*Patients with acutely decompensated cirrhosis were classified as having the AD-1, AD-2, or AD-3 phenotype or having ACLF according to the EASL-CLIF Consortium criteria (1, 2). Data are shown as means ± SD or median (range)*.

*P-values were calculated by unpaired Students' t-test or Kruskall-Wallis test where appropriate*.

*HCV denotes hepatitis C virus; IQR interquartile range; TNF, umor necrosis factor; IL, interleukin; MCP-1, monocyte chemotactic protein 1; IP-10, 10 kDa interferon gamma-induced protein; MIP-1ß, macrophage inflammatory protein 1-beta; G-CSF, granulocyte colony-stimulating factor; GM-CSF, granulocyte-macrophage colony-stimulating factor; IL-1ra, interleukin-1 receptor antagonist protein; IFN, interferon; HNA2, human non-mercaptalbumin 2*.

Interestingly, the pattern of elevated markers for patients in AD-2 and AD-3 were opposite to each other, i.e., markers that were elevated in AD-2 were lower in AD-3 and vice-versa (Figure 1). The addition of elevated markers in AD-2 with the elevated markers in AD-3, recapitulated the profile of systemic inflammation seen in ACLF (Figure 1).

Importantly, not only the distribution of elevated biomarkers, but also the quantitative changes in their levels defined their affiliation to either AD-1, AD-2, or AD-3 (Figure 1, Table 1). Another interesting finding was that patients with ACLF did not show the highest levels of the single markers, but the highest number of elevated markers (Figure 1), suggesting a “full-blown” systemic inflammation in this group of patients and a rather attenuated systemic inflammation in the groups of patients without ACLF.

**Figure 2:**
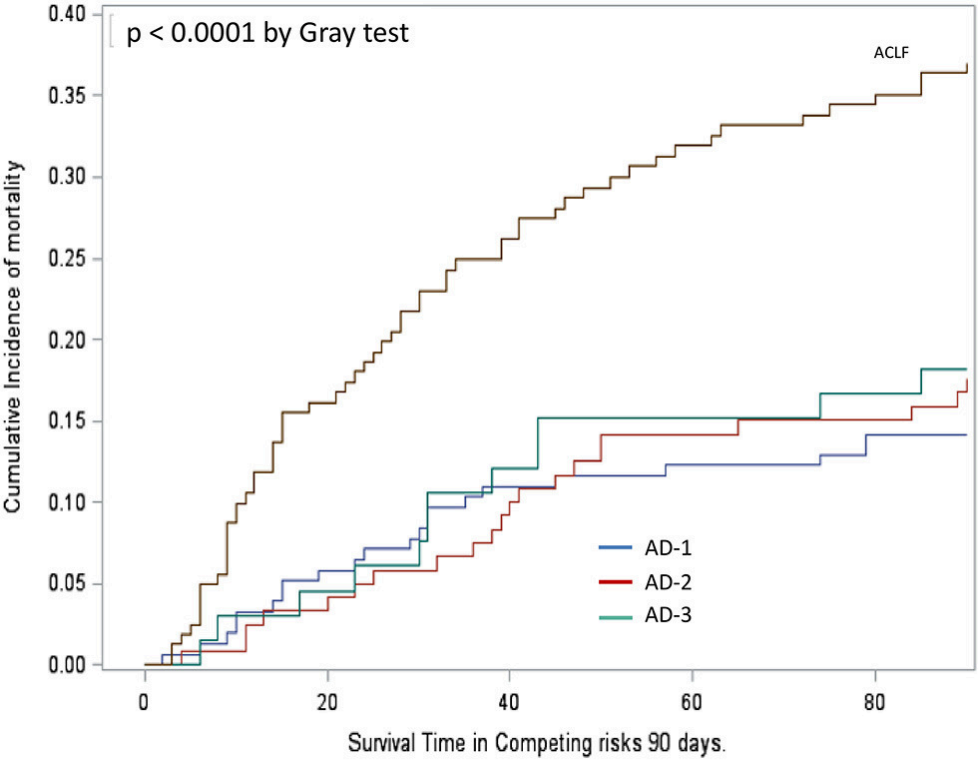
Cumulative incidence function assessing survival in patients' groups analyzed in Figure 1. Mortality was significantly higher in patients with ACLF than in those without, irrespective of their phenotype, AD-1, AD-2, or AD-3 (Gray's test *p* < 0.0001). Mortality did not significantly differ between the three phenotypes AD-1, AD-2, and AD-3. For definitions of these phenotypes, see Figure 1 legend.

Another important observation was that despite the significant differences between the severity and profile of systemic inflammation markers across the three clinical phenotypes of “ACLF-free” AD cirrhosis, the cumulative incidence of death by 90 days, was similar irrespective of the phenotype (Figure 2). In contrast, the “full-blown” systemic inflammation observed in patients with ACLF was associated with increased cumulative incidence of death by 90 days (Figure 2).

### Predicting ACLF Development Using Baseline Systemic Inflammation Profiles

**Figure 3:**
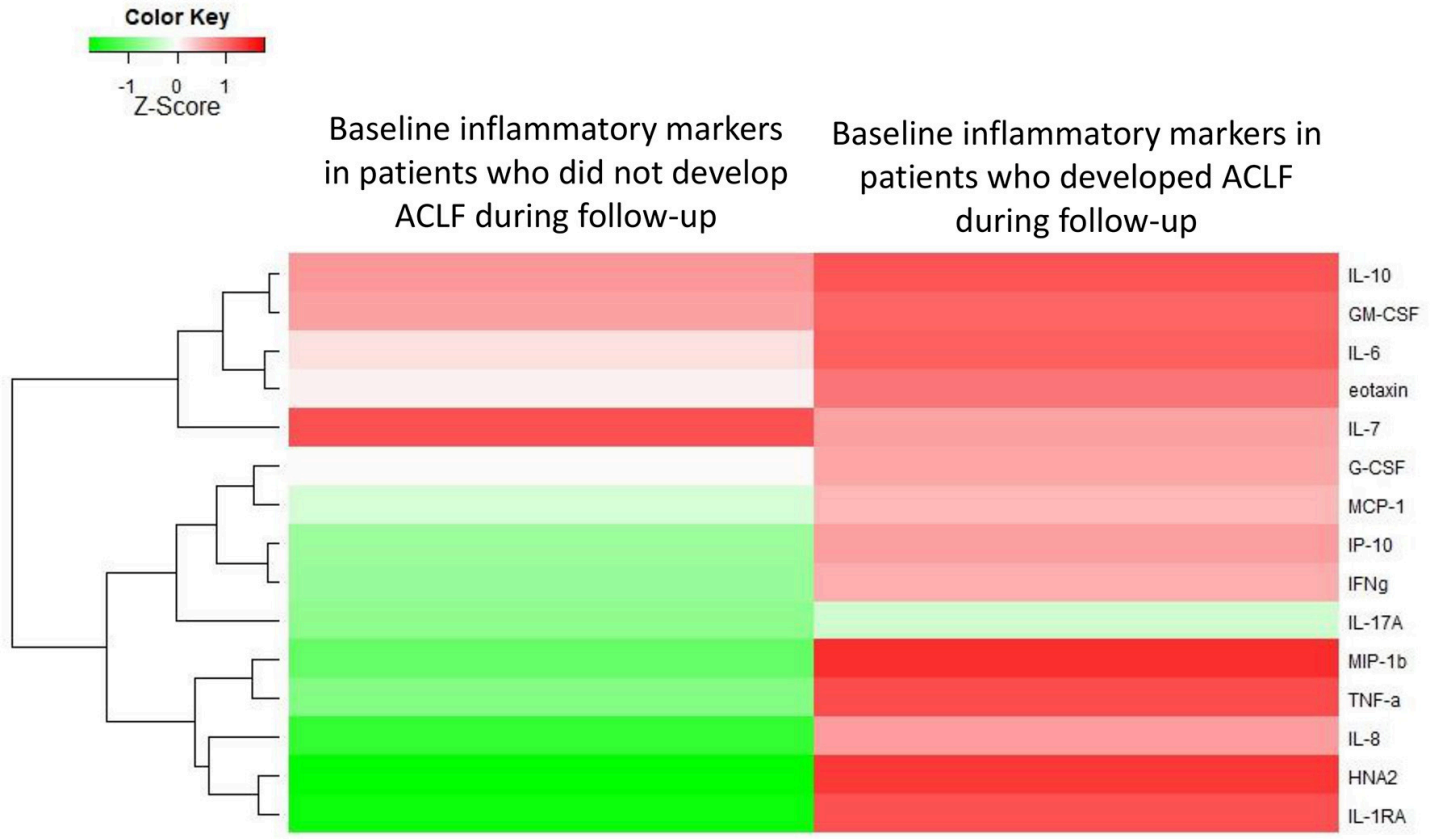
Heat-map showing the median levels of systemic inflammation markers at enrollment of patients with acutely decompensated cirrhosis who were free of ACLF. For the comparison, patients were divided into two groups according to their outcome (i.e., development of ACLF or not, during 28 days of follow-up). The magnitude of the levels is color-coded and the clustering for each marker with the rest of the markers is shown to the left of the heat-map.

Next, we asked whether among AD-patients without ACLF at admission, the baseline systemic inflammation profile differed between those who will subsequently develop ACLF relative to those who will not develop this syndrome. Among the 342 patients with AD at admission, 57 developed ACLF within 28 days after admission. Importantly, baseline levels of systemic inflammation markers were significantly higher among patients who subsequently developed ACLF than among those who remained free of ACLF during the 28-day follow-up (Figure 3, Table 2). Therefore, in AD-patients without ACLF at admission, the development of ACLF can be predicted using the baseline profile of systemic inflammation-related markers.

**Table 2:** Baseline characteristics of 342 patients with acutely decompensated cirrhosis who were free of ACLF at enrollment, divided into two groups according to the development or the absence of development of ACLF during 28 days of follow-up.

**Characteristic**	**Without ACLF development (*N* = 285)**	**With ACLF development (*N* = 57)**	***P*–value**
Age— year	56.7 ± 11.63	58.5 ± 11.00	0.2847
Male gender — no./total no. (%)	191/285 (67.0)	36/57 (63.2)	0.5734
Mean arterial pressure — mm Hg	83.8 ± 11.84	84.2 ± 10.46	0.8259
**PRECIPITATING EVENTS — NO./TOTAL NO. (%)**			
Alcohol consumption	33/265 (12.5)	7/50 (14.0)	0.7631
Mortality 90 days	29/285 (10.2)	26/57 (45.6)	< 0.0001
**ETIOLOGY OF CIRRHOSIS — NO./TOTAL NO. (%)**			
Alcoholic	128/269 (47.6)	29/52 (55.8)	0.2797
HCV	66/269 (24.5)	12/52 (23.1)	0.8224
Alcohol + HCV	26/269 (9.7)	3/52 (5.8)	0.5957
Others	49/269 (18.2)	8/52 (15.4)	0.6248
**MEDIAN VALUES FOR LABORATORY VARIABLES (IQR)**			
Serum albumin—g/dl	2.9 (2.60–3.30)	2.7 (2.19–3.01)	0.0049
Serum bilirubin—mg/d	3.0 (1.60–6.70)	4.4 (1.69–9.06)	0.0963
Serum creatinine—mg/dl	0.9 (0.70–1.32)	1.1 (0.80–1.40)	0.1285
C-reactive protein—mg/L	16.2 (6.00–39.60)	25.8 (10.00–45.60)	0.0887
International Normalized Ratio	1.5 (1.27–1.70)	1.7 (1.42–1.94)	0.0004
Platelets—x10^9^/L	87.0 (57.00–139.00)	94.5 (57.50–119.50)	0.9581
White-cell count—x10^9^/L	6.1 (4.08–9.03)	7.3 (5.04–10.53)	0.0146
**MEDIAN VALUES FOR INFLAMMATORY MEDIATORS (IQR)**			
TNF-α—pg/ml	19.5 (14.14–27.37)	26.7 (17.69–36.52)	< 0.001
IL-6—pg/ml	21.2 (11.27–41.32)	34.3 (19.85–105.54)	< 0.001
IL-8—(pg/ml)	37.3 (20.45–76.41)	58.7 (40.89–108.34)	< 0.001
MCP-1—pg/ml	318.0 (228.03–436.02)	360.0 (276.53–586.47)	0.013
IP-10—pg/ml	965.2 (557.62–1676.00)	1272.0 (760.31–2150.00)	0.053
MIP-1ß—pg/ml	20.1 (13.14–33.55)	36.9 (23.88–56.10)	< 0.001
G-CSF—pg/ml	22.6 (11.19–49.93)	31.0 (15.86–69.19)	0.048
GM-CSF—pg/ml	4.7 (1.96–9.48)	10.7 (3.76–20.05)	< 0.001
IL-10—pg/ml	3.4 (1.12–9.15)	8.0 (2.45–26.42)	0.002
IL1-ra—pg/ml	9.9 (4.72–22.47)	24.0 (12.81–62.11)	< 0.001
IFNγ—pg/ml	5.5 (2.00–18.11)	9.2 (3.20–31.34)	0.013
Eotaxin—pg/ml	110.4 (80.76–155.42)	135.8 (94.97–186.19)	0.008
IL-17A—pg/ml	3.7 (1.57–10.25)	3.4 (1.98–24.37)	0.214
IL-7—pg/ml	2.6 (0.99–8.50)	5.1 (1.88–14.52)	0.032
HNA2—%	4.5 (2.50–8.82)	8.1 (4.89–9.95)	< 0.001

*Patients with acutely decompensated cirrhosis were classified as being free of ACLF or having ACLF according to the EASL-CLIF Consortium criteria (1, 14). Data are shown as means ± SD or median (range). P-values were calculated by unpaired Students' t-test or Man-Whitney U-test where appropriate*.

*HCV, hepatitis C virus; IQR, interquartile range; TNF, umor necrosis factor; IL, interleukin; MCP-1, monocyte chemotactic protein 1; IP-10, 10 kDa interferon gamma-induced protein; MIP-1ß, macrophage inflammatory protein 1-beta; G-CSF, granulocyte colony-stimulating factor; GM-CSF, granulocyte-macrophage colony-stimulating factor; IL-1ra, interleukin-1 receptor antagonist protein; IFN, interferon; HNA2, human non-mercaptalbumin 2*.

When observing the magnitude of specific markers among patients with AD cirrhosis who were free of ACLF on admission, we saw that higher baseline levels of IL-6 (OR, 1.43; 95% CI, 1.04–1.96; *p* = 0.03), IL-1ra (OR, 1.46; 95%-CI 1.10–1.93; *p* = 0.009) and HNA2 (OR, 2.84; 95%-CI 1.52–5.34; *p* = 0.001) were independently associated with development of ACLF within 28 days.

### Baseline Profiles Predicting Survival in Patients With “ACLF-free” AD cirrhosis

**Figure 4:**
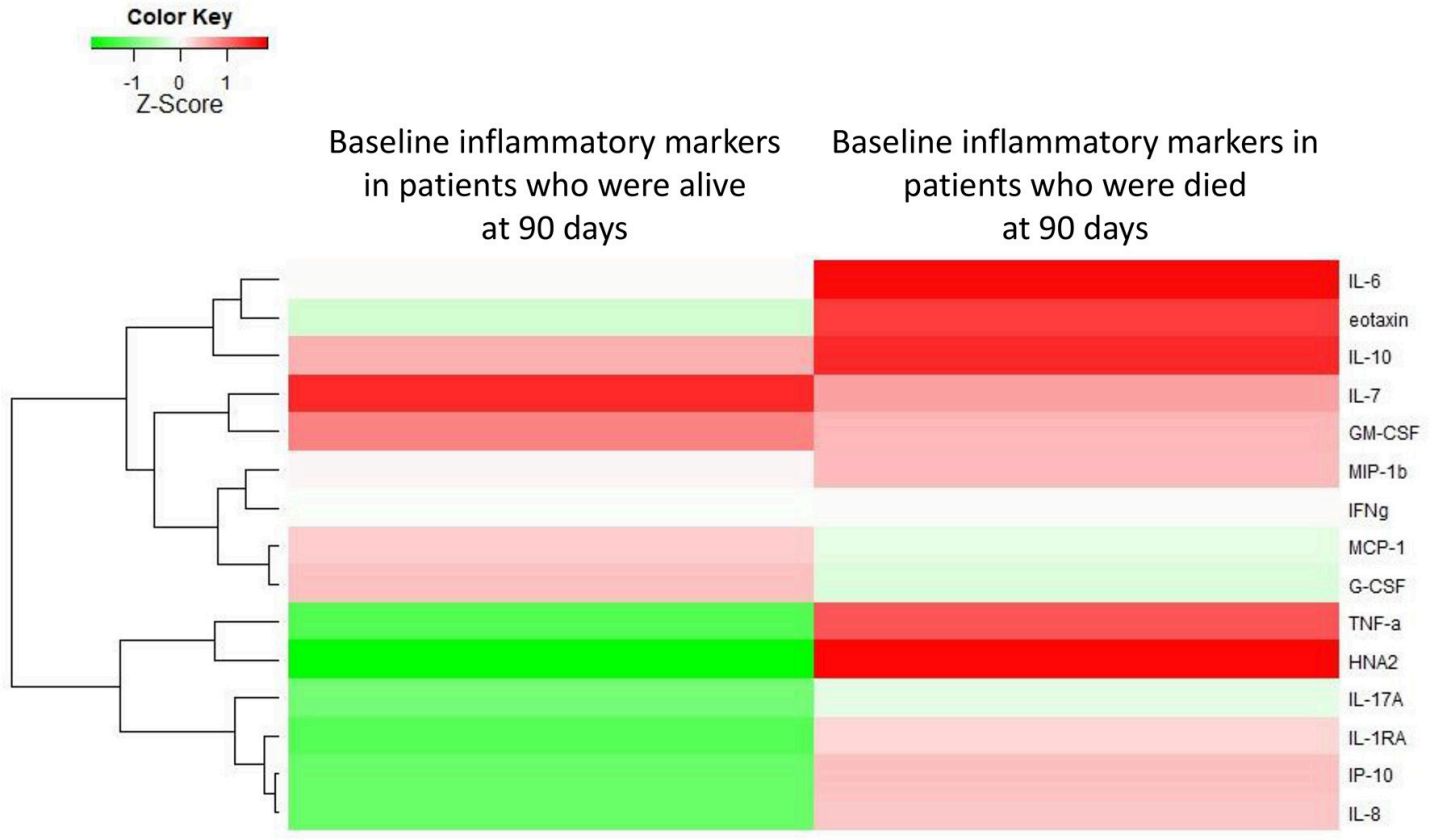
Heat-map showing the median levels of systemic inflammation markers at enrollment of patients with acutely decompensated cirrhosis who were free of ACLF. For the comparison, patients were divided into two groups according to their outcome (i.e., occurrence of death or not during 90 days of follow-up). The magnitude of the levels is color-coded and the clustering for each marker with the rest of the markers is shown to the left of the heat-map.

Among AD-patients without ACLF at hospital admission 55 died and 28 received a liver transplant. The baseline levels of several markers were significantly higher in patients who subsequently died than in those patients who survived (Supplementary Table 4; Figure 4). In particular, TNF-α, IL-6, IL-8, IL-10, eotaxin, IL-17A, IL-7, and HNA2 were higher in patients who died (Supplementary Table 4). Nevertheless, only IL-8 and HNA2 were independently associated with mortality in the patients with AD at baseline (Table 3).

**Table 3:** Hazard ratios for death at 90 days in univariate and multivariable analyses of inflammatory mediators assessed at enrolment of 342 patients with acutely decompensated cirrhosis who were free of ACLF.

**Inflammatory mediator**	**Univariate analysis**			**Multivariate analysis**		
	**Hazard ratio**	**95% IC**	***P*-value**	**Hazard ratio**	**95% IC**	***P*-value**
Tumor necrosis factor-α	1.650	(1.056–2.578)	0.028	–	–	–
Interleukin-6	1.389	(1.094–1.764)	0.007	–	–	–
Interleukin-8	1.521	(1.256–1.842)	< 0.001	1.608	(1.304–1.982)	< 0.001
Eotaxin	2.765	(1.392–5.492)	0.004	–	–	–
Interleukin-17A	1.217	(1.033–1.434)	0.019	–	–	–
Interleukin-7	1.221	(1.047–1.422)	0.011	–	–	–
Human non-mercaptalbumin 2	2.116	(1.456–3.074)	< 0.001	2.237	(1.506–3.323)	< 0.001

*The final models were fitted using a stepwise forward method based on likelihood ratios with the same significance level (p < 0.05) for entering and dropping variables. IC denotes confidence interval*.

## Discussion

This study offers a homogeneous classification way in the heterogeneous population of patients with acutely decompensated cirrhosis, which is related to ACLF development and death. Beyond the large sample size, our study provides novel information by showing that each of the three distinct clinical phenotypes, which compose the group of patients with AD cirrhosis who were free of ACLF had a distinctive baseline inflammatory profile. A second aspect of the novelty in our results is that, among the patients with AD cirrhosis without ACLF, the baseline inflammatory profile was able to distinguish those who will develop ACLF during follow-up from who will not. Finally, we showed that among the patients with AD cirrhosis without ACLF, the baseline inflammatory profile was different between those who will die at short-term and those who will survive.

This novel point of view is demonstrated in four major findings of the present study discussed in the following. The first was that inflammatory markers were only slightly altered in patients with compensated cirrhosis and no prior episode of decompensation. This finding is surprising and interesting considering that many of these patients had clinical significant portal hypertension, as assessed either by the presence of esophageal varices and/or high liver stiffness and low platelets (15). By contrast, most inflammatory mediators were markedly increased in patients admitted to hospital with AD (with or without ACLF). Indeed, this observation is of importance since it shows that severe systemic inflammation and acute decompensation of cirrhosis are concomitant processes, as proposed in the so-called “Systemic Inflammation Hypothesis” (16). This novel finding is probably a result of the careful review of the medical history of the patients included in the compensated control group, excluding any patients with compensated cirrhosis who had prior history of AD episodes. Although it remains unclear which of these processes (acute decompensation or severe systemic inflammation) occurs first, it is tempting to assume that systemic inflammation is a prerequisite for the development of AD cirrhosis. In any case, our findings suggest that systemic inflammation may serve to classify the stage of disease in patients with cirrhosis.

The second important observation was that patients with AD but without ACLF at admission had a very heterogeneous profile of circulating inflammatory mediators. There were three distinct clinical phenotypes (AD-1, AD-2, and AD-3) characterizing those AD patients; each phenotype being associated with distinct profile of systemic inflammation, irrespective of the fact that infection was present or not. The patients hospitalized with AD cirrhosis and neither OF, renal dysfunction nor cerebral dysfunction (AD-1 phenotype), had very mild systemic inflammation, while the patients with an isolated non-renal OF (AD-3 phenotype), and those with isolated renal and/or cerebral dysfunction (AD-2 phenotype) had a higher number of markedly increased markers of systemic inflammation. Moreover, our results obtained in patients with “ACLF-free” AD cirrhosis, suggest a potential explanation for the systemic inflammation signature of ACLF, which can be seen as a result of continuum of activation of systemic inflammation. Indeed, according to the EASL-CLIF consortium definition, the combination of any single non-renal, non-cerebral OF with renal and/or cerebral dysfunction defines ACLF grade 1. While some markers of inflammation were elevated in patients with AD-3 phenotype, other markers were elevated in patients with AD-2 phenotype. As suggested by Figure 1, the profile of systemic inflammation in ACLF could be seen as merging of the inflammatory profile of the AD-2 phenotype and that of the AD-3 phenotype. It could be argued that the division of ACLF-free AD cirrhosis into three phenotypes was arbitrary, one phenotype (AD-2) being more severe than the two others. However, several features do not support this contention. First, the rationale for dividing this group of patients into three distinct phenotypes was based on clinical evidence provided by the CANONIC study (1). Second, the results of the present study now provide a biological support to this distinction by showing that each clinical phenotype was associated with a specific inflammatory profile. Third, none of these inflammatory profiles was as intense as the profile found in patients with ACLF. It was also interesting that, although marked differences in systemic inflammation profiles existed between the three clinical phenotypes of “ACLF-free” AD cirrhosis, there were no significant differences in survival between these three phenotypes. Together these findings indicate that the division into 3 phenotypes was not arbitrary, and more importantly did not underestimate the severity of one phenotype, in particular of the AD-2 phenotype. Our data are novel and very important, indicating that not a maximum level of a specific biomarker, but rather the extension (number of elevated markers) of systemic inflammation, such as that observed in ACLF, must be reached to determine increased mortality. Yet further studies are required to refine the risk assessment in these phenotypes.

There were, however, some differences in the pattern of systemic inflammation across the three clinical phenotypes of “ACLF-free” AD cirrhosis. For example, the presence of an isolated renal and/or cerebral dysfunction was independently associated with high TNF-α levels, while an isolated single non-renal OF was associated with low TNF-α levels. The reasons for these between-group differences in TNF-α expression are unclear but may explain some interesting observations of prior studies. Thus, large-scale trials in severe alcoholic hepatitis showed that anti-TNF approaches (e.g., pentoxifylline) might not work in patients with severe disease and liver failure, but had positive effects in the presence of renal failure (17, 18). Pentoxifylline has also been shown to improve outcomes in patients with alcoholic hepatitis and hepato-renal syndrome (19, 20).

In our study, some markers (i.e., TNF-alpha, HNA2, and IP-10) were elevated in AD-2 patients and lower in AD-3 patients, features, which could seem counterintuitive. We have no clear explanation for these differences, which contribute to the fact that each clinical phenotype has a specific inflammatory profile. We can only speculate on the pathophysiological consequences of differences in some marker levels. For example, TNF-alpha is known to protect the liver by stimulating liver regeneration (21). Therefore, increased TNF-alpha levels in AD-2 may be involved in the absence of liver failure in this group, and, conversely, low TNF-alpha levels could favor the development of liver failure in AD-3 patients. Obviously, future studies are needed. Surprisingly, TNF-alpha and MCP-1 levels were both lower in patients with compensated cirrhosis than in healthy subjects. We do not have clear explanations for these differences, only hypotheses. As mentioned earlier, TNF-alpha is known to stimulate liver regeneration. Therefore, low TNF-alpha levels in patients with compensated cirrhosis may reflect an insufficient TNF-alpha production in the liver, playing a role in subclinical liver failure in these patients. Regarding MCP-1, one should have in mind that this chemokine is produced by damaged tissues to attract monocytes whose function, once migrated, is to restore tissue homeostasis. Therefore, low MCP-1 levels in compensated cirrhosis may result in defective tissue homeostasis.

A third highly relevant finding was the observation that patients with AD cirrhosis who were free of ACLF at enrollment but subsequently developed ACLF within 28 days, had significantly higher baseline levels of inflammatory mediators. Moreover, these patients showed a distinct signature of systemic inflammation, relative to those who did not develop ACLF. These findings reveal that systemic inflammation precedes the development of ACLF, suggesting a cause-to-effect relationship. Importantly, in our study, higher IL-6 levels independently predict ACLF development, a finding which is consistent with previous results showing that elevated IL-6 levels were strongly associated with ACLF and its progression (5). Moreover, higher IL-1ra levels were independently associated with development of ACLF, which is fully in line with previous data demonstrating that polymorphisms of IL-1ra predispose to ACLF (22). Finally, HNA2, a marker for oxidative stress, was independently associated with ACLF development (5, 23). This latter finding calls for an important discussion not only on the pathogenesis of ACLF, but also on the prophylactic treatment since albumin is a potent immune modulator involved in reducing oxidative stress. In fact, there is strong evidence that albumin administration during an episode of spontaneous bacterial peritonitis prevents type I HRS—which represents a special form of ACLF—and improves survival (24). This has also recently been confirmed in the ANSWER trial, a randomized controlled trial in almost 400 patients, showing that long-term weekly albumin administration reduces the incidence of organ failure and thereby improves overall survival in decompensated cirrhotic patients (25). Further studies (e.g., PRECIOSA, NCT03451292) are underway.

Finally, in patients with “ACLF-free” AD cirrhosis, the extension of systemic inflammation at baseline was associated with 90-day mortality. The independent predictors of death were higher levels of IL-8 and HNA2 suggesting that decreasing the levels of these two inflammation-related markers may be an objective for future therapies aiming to increase survival in the group of patients with AD who are at high risk of death. Of note, among patients with AD at enrollment, those who will die had lower G-CSF levels than those who will survive. These patients might benefit from G-CSF therapy as recently shown in patients with ACLF (26).

Although the present study tested a large number of patients and a large number of systemic inflammation mediators, it has its limitations. The concept of this study is to observe systemic inflammation associated with AD cirrhosis (with and without ACLF) without taking into account specific events that could have precipitated the acute decompensation of cirrhosis (e.g., data about use of anti-inflammatory drugs, although unlikely since contraindicated in these patients). Moreover, there might be inter-center heterogeneity in the diagnosis of portal hypertension, although each liver unit participating in the CANONIC study had expertise in the diagnosis and treatment of complications of cirrhosis, therefore limiting the inter-center heterogeneity. Finally, this study did not aim to elaborate on a specific score, but rather to offer pathophysiologic insight into the role of systemic inflammation in patients with AD. Future studies are needed to further elaborate the specific events in AD cirrhosis.

In conclusion, baseline inflammatory markers exhibit no or slight abnormalities in compensated cirrhosis, while in “ACLF-free” AD cirrhosis their profile was heterogeneous, being markedly elevated in those who developed ACLF during follow up. Moreover, among patients with AD cirrhosis who were free of ACLF, this study showed a specific baseline profile of circulating inflammatory mediators in patients who died during follow-up.

## Data Availability

The datasets generated for this study are available on request to the corresponding author.

## Author Contributions

JT, PG, RJ, AG, MB, PA, MP, RM, JC, and VA: study concept and design; JT, AlA, CP, ET, JA-Q, CD, JF-G, SP, PC, KO, JM, ES, WL, MC, TW, CS, RG, TG, MR, and AdG: acquisition of data; JT, AlA, CP, JF-G, KO, JM, RPM, WL, AdG, MP, RM, JC, and VA: analysis and interpretation of data; JT, AlA, CP, AG, RM, JC, and VA: drafting of the manuscript; JT, AlA, CP, JA-Q, CD, SP, PC, MP, and JC: statistical analysis; All authors: critical revision of the manuscript regarding important intellectual content; JT and VA: funding recipient; ET, RoS, SP, RPM, WL, TG, RB, AgA, SZ, VV, FS, FN, CA, AdG, PG, RJ, TS, AG, RuS, MB, PA, RM, JA-Q, and VA: administrative, technical and material support; JT, JF-G, PC, RPM, WL, TG, RB, AgA, SZ, VV, FS, FN, CA, AdG, PG, RJ, TS, AG, RuS, MB, PA, RM, JA, and VA: study supervision.

### Conflict of Interest Statement

The authors declare that the research was conducted in the absence of any commercial or financial relationships that could be construed as a potential conflict of interest.

## Abbreviations

ACLF: acute-on-chronic liver failure
AD: acute decompensation
ADH: antidiuretic hormone
ALT: alanine aminotransferase
BUN: blood urea nitrogen
BT: bacterial translocation
CHE: cholinesterase
G-CSF: granulocyte-colony stimulating factor
GM-CSF: granulocyte-macrophage colony-stimulating factor
HE: hepatic encephalopathy
HNA2: human non-mercaptalbumin-2
HRS: hepatorenal syndrome
HPLC: high performance liquid chromatography
IL: interleukin
IL-1ra: IL-1 receptor antagonist
INFγ: interferon gamma
INR: international normalized ratio
IP-10 (CXCL10): 10kDa interferon gamma-induced protein (C-X-C-motif chemokine 10)
MCP-1 (CCL2): monocyte chemotactic protein 1 (C-C-motif chemokine 2)
MELD: model for end-stage liver disease
MIP-1β: macrophage inflammatory protein 1-beta
NASH: non-alcoholic steatohepatitis
PBC: primary biliary cirrhosis
SD: standard deviation
SEM: standard error of the mean
SI: systemic inflammation
TNFα: tumor necrosis factor alpha.
