# Supplementary material for: Addressing Profiles of Systemic Inflammation Across the Different Clinical Phenotypes of Acutely Decompensated Cirrhosis
Source: Front Immunol. 2019 Mar 19;10:476. doi: 10.3389/fimmu.2019.00476 (PMC6434999; doi:10.3389/fimmu.2019.00476)
Supplement: Supplementary file 1 [file Table_1.docx]

**Supplementary Appendix**

This appendix has been provided by the authors to give readers additional information about their work.

**Table of contents**

|  |  | **Pages** |
| --- | --- | --- |
| **List of investigators and affiliations** |  | **2** |
| **Supplementary Table 1** | **Demographic characteristics and inflammatory mediators in healthy subjects and patients with compensated cirrhosis** | **4** |
| **Supplementary Table 2** | **Clinical characteristics, routine laboratory tests, and inflammatory mediators at enrollment of patients with compensated cirrhosis, patients with acutely decompensated (AD) cirrhosis who were free of ACLF, and patients with ACLF** | **5** |
| **Supplementary Table 3** | **Presence of specific organ dysfunction or organ failure in the patients of the subgroups AD-2 and AD-3** | **7** |
| **Supplementary Table 4** | **Clinical characteristics, routine laboratory tests, and inflammatory mediators at enrollment of 342 patients with acutely decompensated cirrhosis who were free of ACLF, according to their outcome (alive or dead) at 90 days** | **8** |
| **References** |  | **10** |

**List of Investigators**

Jonel Trebicka^1, 2, 3, 4#^, Alex Amoros^1^, Carla Pitarch^1^, Esther Titos^5^, José Alcaraz-Quiles^5^, Carmen Deulofeu^1^, Javier Fernandez-Gomez^6^, Salvatore Piano^7^, Paolo Caraceni^8^, Karl Oettl^9^, Elsa Sola^6^, Wim Laleman^10^, Jane McNaughtan^11^, Rajeshwar P. Mookerjee^11^, Minneke J Coenraad^12^, Tania Welzel^13^, Christian Steib^14^, Rita Garcia^15^, Thierry Gustot^16^, Miguel Angel Rodriguez Gandia^17^, Rafael Bañares^15^, Agustin Albillos^17^, Stefan Zeuzem^13^, Victor Vargas^18^, Faouzi Saliba^19^, Frederic Nevens^10^, Carlo Alessandria^20^, Andrea de Gottardi^21^, Heinz Zoller^22^, Pere Ginès^6^, Rajiv Jalan^11^, Tilman Sauerbruch^2^, Alexander Gerbes^14^, Rudolf Stauber^9^, Mauro Bernardi^8^, Paolo Angeli^7^, Marco Pavesi^1^, Richard Moreau^1,23^, Joan Clària^1,5^ and Vicente Arroyo^1^ on behalf of the CANONIC Study Investigators of the EASL-CLIF Consortium and the European Foundation for the Study of Chronic Liver Failure (EF-CLIF)

**Affiliations:**

^1^ European Foundation for the Study of Chronic Liver Failure, Barcelona, Spain;

^2^ Department of Internal Medicine I, University of Bonn, Germany;

^3^ Faculty of Health Sciences, University of Southern Denmark, Odense, Denmark;

^4^ Institute for Bioengineering of Catalonia, Barcelona, Spain;

^5^Department of Biochemistry and Molecular Genetics, Hospital Clínic, IDIBAPS and CIBERehd Barcelona, Spain;

^6^Liver Unit, Hospital Clínic, IDIBAPS and CIBERehd Barcelona, Spain;

^7^Unit of Internal Medicine and Hepatology, Dept. of Medicine, DIMED, University of Padova, Italy;

^8^Department of Medical and Surgical Sciences, University of Bologna, Bologna, Italy;

^9^Medical University of Graz, Graz, Austria;

^10^University Hospital Gasthuisberg, KU Leuven, Belgium;

^11^Royal Free Hospital, London, UK;

^12^Department of Gastroenterology and Hepatology, Leiden University Medical Center, Leiden, The Netherlands;

^13^J.W. Goethe University Hospital, Frankfurt, Germany;

^14^Department of Medicine II, University Hospital LMU Munich, Liver Center Munich, Munich, Germany;

^15^Department of Digestive Diseases and CIBEREHD, Hospital General Universitario Gregorio Marañón, Instituto de Investigación Sanitaria Gregorio Marañón, and Facultad de Medicina, Universidad Complutense, Madrid, Spain;

^16^Erasme Hospital, Université Libre de Bruxelles, Brussels, Belgium;

^17^Hospital Ramón y Cajal, Madrid, Spain;

^18^ Vall´d Hebron Hospital, Barcelona, Spain;

^19^Hôpital Paul Brousse, Université Paris-Sud, Villejuif, France;

^20^Division of Gastroenterology and Hepatology, San Giovanni Battista Hospital, Torino, Italy;

^21^Department of Hepatology, Inselspital, Bern, Switzerland;

^22^Department of HEpatology and Gastroenterology, University Clinic Innsbruck, Austria;

^23^Inserm, U1149, Centre de Recherche sur l’Inflammation (CRI), UMRS1149; Université Paris Diderot-Paris 7, Département Hospitalo-Universitaire (DHU) UNITY; Service d’Hépatologie, Hôpital Beaujon, Assistance Publique-Hôpitaux de Paris; Laboratoire d’Excellence Inflamex, ComUE Sorbonne Paris Cité, Paris, France;

**^#^Corresponding author:** Jonel Trebicka MD, PhD, European Foundation for the Study of Chronic Liver Failure, Travesera de Gracia 11, 7^th^ floor, 08021 Barcelona, Spain. [jonel.trebicka@efclif.com](mailto:jonel.trebicka@efclif.com)

**Supplementary Table 1. Demographic characteristics and inflammatory mediators in healthy subjects and patients with compensated cirrhosis**

| **Variable** | **Healthy Subjects**  **(N=40)** | **Patients with Compensated Cirrhosis (N=39)** | **P-value** |
| --- | --- | --- | --- |
| **Demographic characteristics** |  |  |  |
| Age— year | 52.4 ± 7.28 | 59.6 ± 9.19 | <0.001 |
| Male gender — no./total no. (%) | 14/20 (70.0) | 23/39 (59.0) | 0.407 |
| **Median values for inflammatory mediators (IQR)** |  |  |  |
| TNF-α — pg/ml | 9.0 (6.52- 11.70) | 6.0 (2.85- 9.46) | 0.001 |
| IL-6 — pg/ml | 0.3 (0.30- 0.30) | 2.4 (0.23- 5.59) | 0.007 |
| IL-8 — pg/ml) | 1.6 (0.64- 3.33) | 5.6 (4.14- 12.54) | <0.001 |
| MCP-1 — pg/ml | 337.1 (217.14- 417.05) | 213.7 (154.6- 276.3) | <0.001 |
| IP-10 — pg/ml | 328.4 (234.61- 432.04) | 484.0 (350.2- 905.9) | 0.002 |
| MIP-1ß — pg/ml | 12.5 (6.38- 17.18) | 11.9 (6.59- 30.96) | 0.384 |
| G-CSF — pg/ml | 2.1 (1.80- 11.04) | 9.7 (2.37- 16.33) | 0.084 |
| GM-CSF — pg/ml | 7.5 (7.50- 7.50) | 2.1 (0.63- 6.86) | 0.007 |
| IL-10 — pg/ml | 1.1 (0.40- 1.10) | 2.7 (1.21- 6.72) | <0.001 |
| IL1-ra — pg/ml | 6.5 (2.72- 8.74) | 7.4 (0.75- 61.61) | 0.958 |
| IFNγ — pg/ml | 0.8 (0.80- 5.46) | 4.4 (0.77- 11.10) | 0.258 |
| Eotaxin — pg/ml | 93.5 (54.14- 125.86) | 81.7 (64.28- 107.51) | 0.345 |
| IL-17A — pg/ml | 0.7 (0.70- 2.98) | 0.9 (0.60- 1.67) | 0.822 |
| IL-7 — pg/ml | 1.4 (1.40- 1.40) | 2.6 (1.93- 3.02) | <0.001 |
| HNA2 — % | 1.3 (0.30- 1.93) | 6.7 (5.51- 7.42) | <0.001 |

NOTE: Data are shown as means ± SD or median (range). P values were calculated by unpaired Students’ t-test or Man-Whitney *U* test where appropriate.

IQR denotes interquartile range; TNF, umor necrosis factor; IL, interleukin; MCP-1, monocyte chemotactic protein 1; IP-10, 10 kDa interferon gamma-induced protein; MIP-1ß, macrophage inflammatory protein 1-beta; G-CSF, **granulocyte colony-stimulating factor;** GM-CSF, **granulocyte-macrophage colony-stimulating factor**; IL-1ra, **interleukin-1 receptor antagonist protein**; IFN, interferon; HNA2, human nonmercaptalbumin 2.

**Supplementary Table 2. Clinical characteristics, routine laboratory tests, and inflammatory mediators at enrollment of patients with compensated cirrhosis, patients with acutely decompensated (AD) cirrhosis who were free of ACLF, and patients with ACLF**

| **Variable** | **Compensated Cirrhosis**  **(N=39)** | **AD Cirrhosis Free of ACLF**  **(N=342)** | **ACLF**  **(N=161)** | **P-value** |
| --- | --- | --- | --- | --- |
| **Clinical characteristics** |  |  |  |  |
| Age— year | 59.6 ± 9.19 | 57.0 ± 11.53 | 57.3 ± 11.45 | 0.405 |
| Male gender — no./total no. (%) | 23/39 (59.0 ) | 227/342 (66.4 ) | 108/161 (67.1 ) | 0.618 |
| Mean arterial pressure — mm Hg | 100.7 ± 11.89 | 83.9 ± 11.61 | 80.8 ± 13.40 | <.001 |
| Etiology of cirrhosis — no./total no. (%) |  |  |  |  |
| Alcoholic | 8/39 (20.5 ) | 157/321 (48.9) | 90/152 (59.2) | <.001 |
| HCV | 17/39 (43.6 ) | 78/321 (24.3) | 27/152 (17.8) | 0.003 |
| Alcohol + HCV | 0/39 (0.0) | 29/321 (9.0) | 17/152 (11.2) | 0.093 |
| Others | 14/39 (35.9) | 57/321 (17.8) | 18/152 (11.8) | 0.002 |
| **Median values for routine laboratory tests (IQR)** |  |  |  |  |
| Serum albumin — g/dl | 4.0 (3.80- 4.40) | 2.9 (2.50- 3.20) | 3.0 (2.40- 3.40) | <.001 |
| Serum bilirubin — mg/d | 0.9 (0.70- 1.17) | 3.1 (1.60- 6.98) | 6.1 (2.00- 14.37) | <.001 |
| Serum creatinine — mg/dl | 0.7 (0.65- 0.89) | 0.9 (0.70- 1.37) | 2.2 (0.98- 3.04) | <.001 |
| C-reactive protein — mg/L | 0.4 (0.13- 0.70) | 18.0 (6.50- 41.00) | 25.0 (9.70- 50.40) | <.001 |
| International Normalized Ratio | 1.1 (1.07- 1.20) | 1.5 (1.30- 1.76) | 1.7 (1.37- 2.30) | <.001 |
| Platelets — x10^9^/L | 108.0 (72.00- 159.00) | 89.0 (57.00- 136.00) | 76.0 (53.00- 121.00) | 0.049 |
| **Supplementary Table 2. (Continued)** |  |  |  |  |
| White-cell count — x10^9^/L | 4.7 (3.65- 7.11) | 6.3 (4.39- 9.40) | 8.0 (5.30- 12.20) | <.001 |
| **Median values for inflammatory mediators (IQR)** |  |  |  |  |
| TNF-α — pg/ml | 6.0 (2.85- 9.46) | 20.2 (14.45- 29.36) | 29.0 (17.38- 42.83) | <.001 |
| IL-6 — pg/ml | 2.4 (0.23- 5.59) | 24.0 (12.22- 47.78) | 36.7 (13.79- 106.83) | <.001 |
| IL-8 — pg/ml) | 5.6 (4.14- 12.54) | 42.4 (22.30- 83.92) | 84.5 (38.64- 165.10) | <.001 |
| MCP-1 — pg/ml | 213.7 (154.6- 276.3) | 324.3 (229.1- 456.3) | 410.3 (293.9- 690) | <.001 |
| IP-10 — pg/ml | 484.0 (350.2- 905.9) | 988.6 (582.3- 1764) | 1147.0 (651.2- 2123) | <.001 |
| MIP-1ß — pg/ml | 11.9 (6.59- 30.96) | 23.3 (13.85- 37.59) | 26.2 (17.89- 42.55) | 0.001 |
| G-CSF — pg/ml | 9.7 (2.37- 16.33) | 23.5 (12.32- 52.01) | 30.5 (13.85- 81.63) | <.001 |
| GM-CSF — pg/ml | 2.1 (0.63- 6.86) | 5.1 (2.19- 11.02) | 6.8 (3.47- 15.97) | <.001 |
| IL-10 — pg/ml | 2.7 (1.21- 6.72) | 3.8 (1.24- 10.79) | 7.2 (1.90- 25.78) | <.001 |
| IL1-ra — pg/ml | 7.4 (0.75- 61.61) | 11.5 (5.20- 28.35) | 18.7 (8.56- 50.48) | <.001 |
| IFNγ — pg/ml | 4.4 (0.77- 11.10) | 6.4 (2.11- 19.73) | 6.0 (2.32- 23.12) | 0.237 |
| Eotaxin — pg/ml | 81.7 (64.28- 107.51) | 113.8 (84.1- 160.5) | 123.5 (86.6- 177.2) | <.001 |
| IL-17A — pg/ml | 0.9 (0.60- 1.67) | 3.6 (1.60- 11.87) | 4.8 (1.62- 14.90) | 0.001 |
| IL-7 — pg/ml | 2.6 (1.93- 3.02) | 2.8 (1.11- 8.93) | 3.5 (1.62- 11.07) | 0.252 |
| HNA2 — % | 6.7 (5.51- 7.42) | 5.2 (2.65- 9.20) | 11.0 (6.25- 15.15) | <.001 |

NOTE: Patients with acutely decompensated cirrhosis were classified as being free of ACLF or having ACLF according to the EASL-CLIF Consortium criteria ^1 2^. Data are shown as means ± SD or median (range). P values were calculated by unpaired Students’ t-test or Kruskall-Wallis test where appropriate.

HCV denotes hepatitis C virus; IQR, interquartile range; TNF, umor necrosis factor; IL, interleukin; MCP-1, monocyte chemotactic protein 1; IP-10, 10 kDa interferon gamma-induced protein; MIP-1ß, macrophage inflammatory protein 1-beta; G-CSF, **granulocyte colony-stimulating factor;** GM-CSF, **granulocyte-macrophage colony-stimulating factor**; IL-1ra, **interleukin-1 receptor antagonist protein**; IFN, interferon; HNA2, human nonmercaptalbumin 2.

**Supplementary Table 3. Presence of specific organ dysfunction or organ failure in the patients of the subgroups AD-2 and AD-3. Data are shown as numbers (%).**

| **Organ function** | **AD-2**  **(N=121)** | **AD-3**  **(N=66)** |
| --- | --- | --- |
| Renal dysfunction | 61(50.4) | 0(0.0) |
| Cerebral dysfunction | 81(66.9) | 0(0.0) |
| Liver failure | 0(0.0) | 38(57.6) |
| Coagulation failure | 0(0.0) | 12(18.2) |
| Respiratory failure | 0(0.0) | 3(4.6) |
| Circulatory failure | 0(0.0) | 5(7.6) |

**Supplementary Table 4. Clinical characteristics, routine laboratory tests, and inflammatory mediators at enrollment of 342 patients with acutely decompensated cirrhosis who were free of ACLF, according to their outcome (alive or dead) at 90 days**

| **Variable** | **Alive at 90 Days**  **(N=259)** | **Dead at 90 days**  **(N=55)** | **P value** |
| --- | --- | --- | --- |
| **Clinical characteristics** |  |  |  |
| Age— year | 57.7 ± 11.50 | 56.6 ± 11.54 | 0.5403 |
| Male gender — no./total no. (%) | 168/259 (64.9) | 36/55 (65.5) | 0.9337 |
| Mean arterial pressure — mm Hg | 84.0 ± 11.86 | 85.4 ± 11.44 | 0.4238 |
| Precipitating events — no./total no. (%) | 31/236 (13.1) | 9/52 (17.3) | 0.4310 |
| Etiology of cirrhosis — no./total no. (%) |  |  |  |
| Alcoholic | 125/246 (50.8) | 23/50 (46.0) | 0.5349 |
| HCV | 58/246 (23.6) | 13/50 (26.0) | 0.7145 |
| Alcohol + HCV | 19/246 (7.7) | 6/50 (12.0) | 0.3992 |
| Others | 44/246 (17.9) | 8/50 (16.0) | 0.7493 |
| **Median values for routine laboratory tests (IQR)** |  |  |  |
| Serum albumin — g/dl | 2.9 (2.59- 3.20) | 2.8 (2.30- 3.20) | 0.1675 |
| Serum bilirubin — mg/d | 2.6 (1.49- 5.67) | 5.2 (2.91- 10.60) | <.0001 |
| Serum creatinine — mg/dl | 0.9 (0.70- 1.28) | 1.0 (0.70- 1.45) | 0.2967 |
| C-reactive protein — mg/L | 15.0 (5.35- 36.30) | 36.5 (18.00- 61.00) | <.0001 |
| International Normalized Ratio | 1.4 (1.24- 1.67) | 1.7 (1.47- 2.03) | <.0001 |
| Platelets — x10^9^/L | 92.0 (58.00- 141.00) | 88.8 (55.00- 122.00) | 0.4115 |
| White-cell count — x10^9^/L | 6.1 (4.10- 8.90) | 8.0 (5.67- 12.56) | 0.0002 |
|  |  |  |  |
| **Supplementary Table 4. (Continued)** |  |  |  |
| **Median values for inflammatory mediators (IQR)** |  |  |  |
| TNF-α — pg/ml | 19.40 (14.14 - 27.33) | 25.35 (17.22 - 32.06) | 0.006 |
| IL-6 — pg/ml | 21.13 (11.72 - 40.71) | 34.14 (17.69 - 80.24) | 0.002 |
| IL-8 — pg/ml) | 37.33 (19.94 - 79.81) | 58.73 (41.69 - 117.82) | <.001 |
| MCP-1 — pg/ml | 323.06 (235.91 - 436.79) | 349.46 (245.99 - 546.44) | 0.196 |
| IP-10 — pg/ml | 972.14 (577.41 - 1722.00) | 1120.00 (580.44 - 1996.00) | 0.565 |
| MIP-1ß — pg/ml | 22.58 (13.92 - 37.50) | 26.25 (15.44 - 45.50) | 0.239 |
| G-CSF — pg/ml | 23.51 (11.70 - 52.24) | 23.79 (15.57 - 46.06) | 0.841 |
| GM-CSF — pg/ml | 4.71 (2.03 - 10.37) | 5.54 (2.57 - 11.91) | 0.121 |
| IL-10 — pg/ml | 3.03 (0.91- 9.75) | 5.93 (1.84 - 16.99) | 0.043 |
| IL1-ra — pg/ml | 10.44 (4.98 - 28.88) | 15.49 (6.35 - 31.87) | 0.116 |
| IFNγ — pg/ml | 6.02 (2.01 - 19.38) | 7.83 (2.78 - 26.79) | 0.370 |
| Eotaxin — pg/ml | 106.75 (77.61 - 152.18) | 137.18 (99.03 - 187.88) | <.001 |
| IL-17A — pg/ml | 3.48 (1.49 - 10.25) | 5.13 (2.21 - 17.41) | 0.030 |
| IL-7 — pg/ml | 2.75 (1.00 - 8.17) | 4.49 (1.63 - 16.41) | 0.041 |
| HNA2 — % | 4.37 (2.38 - 8.17) | 8.35 (5.34 - 11.13) | <.001 |

NOTE: Data are shown as means ± SD or median (range). P values were calculated by unpaired Students’ t-test or Man-Whitney *U* test where appropriate.

Data obtained in 28 patients who received a liver transplant during the 90 days were excluded from this analysis.

HCV denotes hepatitis C virus; IQR interquartile range; TNF, umor necrosis factor; IL, interleukin; MCP-1, monocyte chemotactic protein 1; IP-10, 10 kDa interferon gamma-induced protein; MIP-1ß, macrophage inflammatory protein 1-beta; G-CSF, **granulocyte colony-stimulating factor;** GM-CSF, **granulocyte-macrophage colony-stimulating factor**; IL-1ra, **interleukin-1 receptor antagonist protein**; IFN, interferon; HNA2, human nonmercaptalbumin 2.

**References**

1. Moreau R, Jalan R, Gines P, et al. Acute-on-chronic liver failure is a distinct syndrome that develops in patients with acute decompensation of cirrhosis. *Gastroenterology* 2013;144(7):1426-37, 37 e1-9. doi: 10.1053/j.gastro.2013.02.042

2. European Association for the Study of the Liver. , Collaborators:, Angeli P, et al. EASL Clinical Practice Guidelines for the management of patients with decompensated cirrhosis. *Journal of hepatology* 2018;69(2):406-60. doi: 10.1016/j.jhep.2018.03.024
